# Interrater reliability of the violence risk assessment checklist for youth: a case vignette study

**DOI:** 10.1186/s12888-024-05746-8

**Published:** 2024-04-23

**Authors:** Anniken L. W. Laake, John Olav Roaldset, Tonje Lossius Husum, Stål Kapstø Bjørkly, Carina Chudiakow Gustavsen, Øyvind Lockertsen

**Affiliations:** 1 Faculty of Health Sciences, Department of Nursing and Health Promotion, Oslo Metropolitan University, Oslo, Norway; 2 Oslo University Hospital, Centre for Research and Education in Forensic Psychiatry, Oslo, Norway; 3 Faculty of Health Sciences and Social Care, Molde University College, Molde, Norway; 4 Department of Child and Adolescent Psychiatry, Oslo University Hospital, Oslo, Norway

**Keywords:** Adolescents, Child protective services, Interrater reliability, Risk screening, Violence, Youth psychiatry

## Abstract

**Background:**

Facilities providing health- and social services for youth are commonly faced with the need for assessment and management of violent behavior. These providers often experience shortage of resources, compromising the feasibility of conducting comprehensive violence risk assessments. The Violence Risk Assessment Checklist for Youth aged 12–18 (V-RISK-Y) is a 12-item violence risk screening instrument developed to rapidly identify youth at high risk for violent behavior in situations requiring expedient evaluation of violence risk. The V-RISK-Y instrument was piloted in acute psychiatric units for youth, yielding positive results of predictive validity. The aim of the present study was to assess the interrater reliability of V-RISK-Y in child and adolescent psychiatric units and acute child protective services institutions.

**Methods:**

A case vignette study design was utilized to assess interrater reliability of V-RISK-Y. Staff at youth facilities (*N* = 163) in Norway and Sweden scored V-RISK-Y for three vignettes, and interrater reliability was assessed with the intraclass correlation coefficient (ICC).

**Results:**

Results indicate good interrater reliability for the sum score and Low-Moderate-High risk level appraisal across staff from the different facilities and professions. For single items, interrater reliability ranged from poor to excellent.

**Conclusions:**

This study is an important step in establishing the psychometric properties of V-RISK-Y. Findings support the structured professional judgment tradition the instrument is based on, with high agreement on the overall risk assessment. This study had a case vignette design, and the next step is to assess the reliability and validity of V-RISK-Y in naturalistic settings.

## Background

While evaluations of an individual’s risk for violent behavior were traditionally developed within and for forensic settings [1], the need for assessing violence risk has been identified in additional settings where management of violent behavior commonly occurs [2, 3]. Violence risk assessments are increasingly conducted in multiple contexts of healthcare and other public services [3, 4], including mental healthcare [2] and emergency departments [5, 6]. As the settings in which violence risk assessments are utilized expand, the need emerges for tools tailored to target populations and the evaluation of their psychometric properties.

Institutions providing healthcare and social services for youth commonly face challenges related to aggression and violence. In youth psychiatric units, inpatient violence is a substantial concern, negatively impacting the wellbeing of both patients and staff [7]. For instance, a chart review study in an inpatient psychiatric unit for adolescents showed that aggressive or violent behavior requiring intervention were recorded for 28.4% of inpatients [8]. Further, child protective services (CPS) is identified as one of the public services at highest risk of encountering violent behavior from youth [9]. Thus, these are settings with a need for available resources to identify, prevent and manage violence risk. The ability to accurately identify individuals at high risk for violent behavior facilitates implementation of interventions to prevent violence from occurring [4, 10–12], and aids decision-making related to patient treatment [13, 14]. Further, routinely assessing violence risk in inpatient psychiatric settings can help de-escalate aggressive behavior [15] and can reduce use of coercive measures, promoting a safer environment for patients and staff [16–19].

### Violence risk assessments for youth

For children and adolescents, assessing violence risk require distinct situational and intrapersonal considerations (e.g. school setting; caretaker situation; cognitive developmental stage; psychiatric diagnostics), and assessments for adults can not readily be generalized to youth [1, 20]. Violence risk assessment tools specifically for youth have been developed and validated for various settings, with the Structured Assessment of Violence Risk in Youth (SAVRY) [21] and Youth Level of Service/Case Management Inventory (YLS/CMI) [22] among the most commonly researched [23, 24]. These instruments are comprehensive, time consuming to administer, and may require specific training to utilize [e.g. 25, 26]. In facilities commonly required to make judgments about violence risk, such as emergency departments and acute psychiatric institutions, structural and contextual aspects like scarcity of time and staff resources influence the ability to routinely conduct in-depth violence risk assessments [27, 28].

For adults, several shorter violence risk screeners, such as V-RISK-10 [29], have been designed to rapidly identify individuals at high risk for violence. In situations where comprehensive assessments are not feasible, these brief instruments can be used to quickly identify high-risk individuals and guide initial decisions about the need for more comprehensive risk assessments or implementation of immediate interventions [30, 31]. However, in comparison to adult populations, the development of violence risk screening instruments for youth is lagged. In lieu of available instruments tailored to youth, providers have in some instances used tools designed for adult populations, such as V-RISK-10 [32].

### Violence risk assessment checklist for youth aged 12–18 (V-RISK-Y)

The V-RISK-Y is a violence risk screening tool for youth aged 12 to 18, based on the V-RISK-10 screener. The instrument is designed to be time-efficient, self-explanatory, and possible to use without prior training [32]. As such, it caters to institutions providing 24-hour care, where acute evaluations are conducted, and where staff trained in risk assessment may not readily be available.

V-RISK-Y was piloted in an emergency psychiatric unit for youth in Norway [32]. Results indicated good predictive validity for violent events during the youth’s hospital stay, with Area Under the Curve of the Receiver Operating Characteristics (AUC) of a high value of 0.762 for the sum score of V-RISK-Y. Interrater reliability for V-RISK-Y was assessed in the pilot by scoring of case vignettes. As measured by intraclass correlation coefficient (ICC), interrater reliability for individual items was poor to fair, and the ICC for the sum score was 0.51. The pilot version of V-RISK-Y included relevance scores for each of the 12 items, but as the relevance scores were perceived as confusing by participants it was hypothesized that they contributed to low ICC values [32]. Because V-RISK-Y is a new instrument, there is a need to establish psychometric properties of the screening in relevant settings and with a larger sample size.

### Interrater reliability

While knowledge about interrater reliability is essential for evaluating the validity of a scale, reporting of interrater reliability in psychometric studies is commonly neglected [33]. For violence risk assessment, concordance between participants in assessing violence risk has been associated with predictive validity [34]. McNiel and colleagues (2000) assessed interrater reliability and predictive validity for risk assessments in an inpatient psychiatric unit and found that predictive validity of assessed risk significantly improved with agreement between participants.

Previous studies on interrater reliability for violence risk assessments have commonly utilized the interrater correlation coefficient (ICC) as reliability measure [e.g. 29, 35, 36], which indicates the variation between raters measuring the same individuals [37]. A study on V-RISK-10 assessed interrater reliability in an in-patient psychiatric setting, and included ratings for 73 acute psychiatric patients from 25 participants [29]. ICC values indicated good interrater reliability for the sum score, fair for the Low-Moderate-High risk level, and for single items ICC ranged from poor to good [29]. In a systematic review of psychometric properties of violence risk assessments for youth, ICC estimates for SAVRY and YLS/CMI indicated fair to excellent interrater reliability across published studies [38]. Given the lack of established violence risk screening instruments for youth, interrater reliability of violence risk screeners for youth has not been assessed other than in the V-RISK-Y pilot study.

### Aims

The main objective of the present study was to assess the interrater reliability of V-RISK-Y in facilities where youth receive psychiatric in- and outpatient care, as well as acute child protective services institutions. A secondary aim was to assess whether there are differences in interrater reliability between types of youth facilities, or between types of staff in these institutions.

## Methods

### Design, setting and participants

This study was designed as a case vignette study, where staff from mental health and child protective services (*N* = 163) rated V-RISK-Y for three written cases. Vignettes simulate real life and allows for controlling included variables, ensuring all participant responses are based on the exact same information [39]. The design circumvents ethical and practical challenges which commonly arise when including vulnerable individuals in research [40], and thus is beneficial for initial assessment of an instrument. Generalizability is one of the major criticisms of this methodology as the complexities of real life can be difficult to capture in a written scenario [39, 40].

Participants were recruited among staff from youth facilities participating in an ongoing V-RISK-Y multicenter study in Norway and staff from youth psychiatric units participating at a V-RISK-Y seminar in Sweden. Ethical approval was granted by the Regional Committee for Medical and Health Research Ethics (REK ID: 218444).

Sample characteristics are illustrated in Table 1. Participants included psychologists (*n* = 15), physicians (*n* = 18), as well as staff members with other professions (*n* = 106). Staff other than physicians and psychologists consisted of professions that do not require postgraduate education, including nurses, social workers, social educators, and youth workers. Only staff in direct contact with youth were included (i.e. administrative staff was not included).

**Table 1:** Participant characteristics

	Acute psychiatric units	CPS institutions	Swedish youth psychiatric units	Total
Psychologist	2	-	13	15
Physician	11	-	7	18
Other professions*	38	52	16	106
Missing	21	-	3	24
Total	72	52	39	163

*Includes staff with professions that do not require postgraduate education

The Norwegian youth facilities consisted of four acute psychiatric units (*n* = 72) providing in-patient services, and four acute child protective services (CPS) institutions (*n* = 52) providing residential care. CPS institutions are custodial institutions for acute placement of youth without satisfactory home conditions [41], and staff at these institutions largely consist of social workers and youth workers.

Participants from Sweden included staff from six child and adolescent psychiatric units (*n* = 39). These units consisted of facilities providing outpatient mental health services as well as units for inpatient acute psychiatric care for children and adolescents.

### Materials

Measures consisted of printed copies of V-RISK-Y and three case vignettes. Materials were developed in Norwegian and translated to Swedish in collaboration with Swedish mental health professionals.

#### V-RISK-Y

V-RISK-Y consists of 12 items: V1) Prior and/or current acts of violence; V2) Prior and/or current threats of violence; V3) Prior and/or current alcohol or substance abuse; V4) Prior and/or current severe symptoms of mental health disorders; V5) Disruptive, impulsive behaviour/Behavioural disorder; V6) Poor insight into the mental disorder and/or behaviour; V7) Suspicion; V8) Demonstrates lack of empathy; V9) Unrealistic planning; V10) Future stressful situations; V11) Prior and/or current severe trauma; and V12) The youth and parents/guardians’ perception of risk. Each item is rated according to their presence as “No”, “Moderate/Maybe”, “Yes”, or “Don’t Know”. The level of violence risk, “Low”, “Moderate”, or “High” is categorically indicated by the rater based on item scores combined with clinical judgment, following the structured professional judgment (SPJ) tradition [42]. The relevance scores previously included are removed from the current version. If feasible, the screening should be scored interdisciplinary. It is recommended to do the scoring upon the initial contact with the youth, such as after the intake interview, without the youth or their parents/guardians present.

#### Vignettes

The case vignettes were each approximately one page. Vignette summaries are included in Appendix 1. Cases were developed by clinicians and researchers experienced in violence risk assessment and youth psychiatry, and designed to reflect cases commonly encountered in youth psychiatric units and CPS institutions. While no psychiatric diagnoses were specified or fully described in the cases, the description of Case 1 (Farhad) alludes to autism specter disorder, Case 2 (Peter) describes antisocial behavior and symptoms of behavioral disorders, and Case 3 (Jeanette) indicates a depressive reaction. To reflect a clinical setting where information about youth might be lacking at intake, each case was designed with incomplete information to allow for the “don’t know” response to be an appropriate score for some items.

### Procedure

Researchers visited each youth facility interested in participating and gave an introduction of the development and structure of V-RISK-Y. Because V-RISK-Y is designed to be self-explanatory, no in-depth training in scoring the instrument was provided. Staff who agreed to participate were given writeups of the cases and V-RISK-Y forms and asked to independently rate each case to the best of their ability in one sitting. Ratings were conducted anonymously, without the researchers present. While no specific time limit was given for rating the cases, participants typically spent a total of 15 to 30 min completing the scorings.

### Statistical analyses

Statistical analyses were conducted in Stata Statistical Software 17.0. Statistical significance level was set to 0.05 for all levels. Interrater reliability was assessed by estimating the intraclass correlation coefficient (ICC) for the 12 V-RISK-Y individual items, the sum score, and the risk level (Low-Moderate-High). ICC values range from 0 to 1, and interrater reliability is typically interpreted as low for values below 0.50, moderate for values between 0.50 and 0.75, good for values between 0.75 and 0.90, and excellent for values above 0.90 [37]. Because of clustering of data due to shared environmental and individual factors among participants, ICC was estimated based on multilevel statistical models which account for homogeneity [43], using the *estat icc *command.

The risk level was scored on an ordinal scale, as Low (1), Moderate (2) and High (3). V-RISK-Y items were also interpreted on an ordinal scale as “No” (0), “Don’t Know” (1), “Moderate/Maybe” (2), and “Yes" (3). “Don’t know” ratings were weighted as 1 and included in item analyses, which was also the method for analyses in the recent pilot study on V-RISK-Y [32]. Accounting for their ordinal properties, ICC for these variables was calculated based on multilevel ordered logistic regression [44]. For the sum score variable (range 0–36), mixed linear regression was used to estimate ICC [45].

Analyses were conducted for the overall data, and stratified analyses were conducted for type of institution and type of profession. As there is overlap in responsibilities of psychologists and physicians in the participating facilities, these professions were combined and compared to the other professions to increase statistical power.

For ten submitted forms, the full V-RISK-Y scoring was missing, and these ratings were excluded. All ten excluded ratings were for Case 3, Jeanette. There were no more than three missing values for any included V-RISK-Y ratings. Given the low number of missing scores in the included ratings, as displayed in Table 2, missing values were not replaced.

## Results

Table 2 shows the frequency of scores for each V-RISK-Y item by case. The mean sum score for the cases was 24.43 [SD = 2.74] for Case 1, 27.99 [SD = 3.12] for Case 2, and 16.15 [SD = 5.00] for Case 3.

**Table 2:** Frequencies of V-RISK-Y scores by case

	Case 1: Farhad (*N* = 163)						Case 2: Peter (*N* = 163)					Case 3: Jeanette (*N* = 153)					
	*No*		*Don’t know*	*Maybe/* *Moderate*	*Yes*	*Missing*		*No*	*Don’t know*	*Maybe/* *Moderate*	*Yes*	*Missing*	*No*	*Don’t know*	*Maybe/* *Moderate*	*Yes*	*Missing*
**V1**	1		0	10	152	-		0	0	10	153	-	137	9	5	1	1
**V2**	0		0	4	159	-		3	2	13	145	-	30	7	95	19	2
**V3**	84		74	3	0	2		2	4	69	88	-	48	19	66	17	3
**V4**	10		19	62	68	4		13	36	50	61	3	16	10	59	67	1
**V5**	0		2	21	137	3		1	6	19	136	1	37	11	75	29	1
**V6**	1		8	101	51	2		0	3	19	141	-	44	18	78	10	3
**V7**	49		52	37	18	9		68	72	10	10	3	45	24	57	25	2
**V8**	2		3	10	148	-		1	5	16	141	-	134	6	8	2	3
**V9**	3		19	90	51	-		1	1	22	135	4	45	47	56	3	2
**V10**	8		30	49	74	2		1	8	22	130	2	18	27	74	31	3
**V11**	30		123	5	2	3		28	118	15	1	1	6	11	39	94	3
**V12**	5		21	31	106	-		26	21	53	61	2	68	38	34	12	1
**Risk**	*Low*	*Moderate*			*High*	*Missing*		*Low*	*Moderate*		*High*	*Missing*	*Low*	*Moderate*		*High*	*Missing*
	1	31			115	16		1	16		126	20	124	12		-	17

Results for analyses of interrater reliability for the overall data and stratified analyses for the types of youth facilities are presented in Table 3. For the overall data, interrater reliability is excellent for V1 (Violence), good for V2 (Threats), moderate for V3 (Substance abuse), poor for V4 (Severe mental health symptoms), moderate for V5 (Disruptive behavior), good for V6 (Insight), poor for V7 (Suspicion), good for V8 (Empathy), moderate for V9 (Unrealistic plans) and V10 (Future stress), good for V11 (Trauma) and poor for V12 (Own perception). ICC estimates remained identical when type of institution and profession was controlled for in the mixed model. For stratified analyses, confidence intervals are wide and overlapping for all individual items.

**Table 3:** Intraclass correlation coefficient (ICC) values for V-RISK-Y item scores, sum score, and risk category

	Full sample (*N* = 163)	Acute psychiatric units (*n* = 72)	CPS institutions (*n* = 52)	Swedish youth psychiatric units (*n* = 39)
Item	*ICC [95% CI]*	*ICC [95% CI]*	*ICC [95% CI]*	*ICC [95% CI]*
V1	0.94 [0.76, 0.99]*	0.97 [0.86, 0.99]*	0.99 [0.99, 0.99]*	0.89 [0.58, 0.98]
V2	0.84 [0.51, 0.96]	0.84 [0.49, 0.97]	0.95 [0.67, 0.99]	0.93 [0.48, 1.0]
V3	0.68 [0.30, 0.92]	0.67 [0.28, 0.91]	0.83 [0.47, 0.96]	0.60 [0.22, 0.89]
V4	0.01 [0.00, 0.16]	0.06 [0.01, 0.39]	0.23 [0.05, 0.64]	0.04 [0.00, 0.53]
V5	0.73 [0.35, 0.93]	0.81 [0.45, 0.96]	0.63 [0.23, 0.90]	0.78 [0.38, 0.95]
V6	0.76 [0.38, 0.94]	0.74 [0.35, 0.94]	0.62 [0.24, 0.90]	0.94 [0.75, 0.99]
V7	0.14 [0.03, 0.46]	0.03 [0.00, 0.36]	0.24 [0.05, 0.65]	0.32 [0.07, 0.73]
V8	0.88 [0.58, 0.97]	0.85 [0.53, 0.97]	0.93 [0.72, 0.99]	0.95 [0.59, 1.0]
V9	0.74 [0.36, 0.93]	0.75 [0.37, 0.94]	0.74 [0.35, 0.94]	0.76 [0.37, 0.95]
V10	0.54 [0.19, 0.86]	0.64 [0.25, 0.91]	0.33 [0.08, 0.74]	0.65 [0.25, 0.91]
V11	0.77 [0.40, 0.95]	0.83 [0.47, 0.96]	0.75 [0.36, 0.94]	0.75 [0.35, 0.94]
V12	0.47 [0.15, 0.82]	0.48 [0.15, 0.83]	0.42 [0.12, 0.80]	0.52 [0.17, 0.86]
Sum score	0.81 [0.47, 0.96]	0.83 [0.49, 0.96]	0.75 [0.37, 0.94]	0.86 [0.55, 0.97]
Risk	0.97 [0.71, 1.0]*	0.98 [0.83, 1.0]*	0.96 [0.79, 0.99]	0.99 [0.93, 1.0]

*Convergence not achieved for mixed ordered logistic regression model

### Interrater reliability for types of youth facilities

Interrater reliability is good for V-RISK-Y sum score, and excellent for the Low-Moderate-High risk level across type of facility. For the acute psychiatry group, interrater reliability is excellent for V1, good for V2, moderate for V3, poor for V4, good for V5, moderate for V6, poor for V7, good for V8 and V9, moderate for V10, good for V11, and poor for V12.

For the CPS institutions, interrater reliability is excellent for V1 and V2, good for V3, poor for V4, moderate for V5 and V6, poor for V7, excellent for V8, moderate for V9, poor for V10, good for V11, and poor for V12.

For the Swedish units, interrater reliability is good for V1, excellent for V2, moderate for V3, poor for V4, good for V5, excellent for V6, poor for V7, excellent for V8, good for V9, moderate for V10, good for V11, and moderate for V12.

### Interrater reliability for professional groups

Results for interrater reliability for the professional groups are presented in Table 4.

**Table 4:** Intraclass correlation coefficient (ICC) values for professions

	Physician/ Psychologist (*n* = 33)	Other professions (*n* = 106)
Item	*ICC [95% CI]*	*ICC [95% CI]*
V1	*	0.98 [0.89, 1.0]**
V2	0.99 [0.62, 1.0]	0.91 [0.65, 0.98]
V3	0.62 [0.23, 0.90]	0.74 [0.36, 0.94]
V4	0.00 [-, 1]	0.02 [0.00, 0.23]
V5	0.94 [0.72, 0.99]	0.68 [0.29, 0.92]
V6	0.98 [0.91, 1.0]	0.72 [0.33, 0.93]
V7	0.10 [0.01, 0.51]	0.21 [0.04, 0.59]
V8	*	0.90 [0.64, 0.98]
V9	0.94 [0.71, 0.99]	0.74 [0.35, 0.93]
V10	0.65 [0.25, 0.91]	0.47 [0.15, 0.83]
V11	0.91 [0.61, 0.99]	0.74 [0.36, 0.94]
V12	0.58 [0.20, 0.88]	0.40 [0.11, 0.78]
Sum score	0.86 [0.55, 0.97]	0.79 [0.43, 0.95]
Risk	0.97 [0.78, 1.0]**	0.97 [0.82, 0.99]

* Convergence not achieved for mixed ordered logistic regression model. ICC not estimated

** Convergence not achieved for mixed ordered logistic regression model, but ICC is estimated

Across the professional groups, interrater reliability is good for the sum score and excellent for the Low-Moderate-High risk level. For the physician/psychologist group, ICC values did not compute for items V1 and V8. Interrater reliability is excellent for V2, moderate for V3, poor for V4, excellent for V5 and V6, poor for V7, excellent for V9, moderate for V10, excellent for V11, and moderate for V12.

For the other professions, interrater reliability is excellent for V1 and V2, moderate for V3, poor for V4, moderate for V5 and V6, poor for V7, excellent for V8, moderate for V9, poor for V10, moderate for V11, and poor for V12.

## Discussion

Results indicate overall good interrater reliability for V-RISK-Y, and moderate to good interrater reliability for most individual items. These findings are comparable to interrater reliability for other youth violence risk assessments tools [38], as well as for V-RISK-10 [29], a recommended violence risk screener for adults utilized internationally [31]. There were few differences in interrater reliability between the youth facilities included in the study, which is promising for the potential utility of V-RISK-Y across settings where violence risk screening of youth is needed. No major differences in interrater reliability were found between Swedish and Norwegian units, implying that the level of agreement between staff at youth facilities in Sweden and Norway is similar.

### Sum score and risk level

The interrater reliability for the sum score is consistently high, indicating agreement between participants on the sum of present risk factors presented in the cases. Results indicate good interrater reliability for the sum score of V-RISK-Y, and excellent for the Low-Moderate-High risk level across all types of facilities. Similarly, interrater reliability for the sum score is good, and excellent for the Low-Moderate-High risk level across the professional groups. These results are encouraging, as it indicates that there is overall agreement on the risk level assigned to the cases based on the V-RISK-Y scoring. These results lend support to the SPJ tradition in which V-RISK-Y is developed, demonstrating high agreement of the discretionary risk assessment guided by scoring the instrument [46].

### Single items

For most single items, interrater reliability is consistently moderate to good across all groups. For items representing static risk factors, such as V1 (Violence) and V2 (Threats), interrater reliability is good to excellent. For V3 (Substance abuse), interrater reliability is good for CPS institutions while moderate for the other types of facilities. These items are likely relatively easy to score provided the availability of relevant information.

The poorest interrater reliability is found for V4 (Severe symptoms of mental health disorder), where ICC is close to zero across all groups. In the V-RISK-Y pilot study, which also assessed interrater reliability with case vignettes, the ICC value of 0.66 for V4 indicates moderate interrater reliability [32]. Characteristics of the case vignettes could provide one possible explanation of the low reliability measure for this item. Few typical symptoms of mental health disorders were described in the cases, and there was no mention of previous or current psychiatric diagnoses. The first case of Farhad describes a condition that could be compatible with autism specter disorder, where challenges in communication and social interactions are highlighted. For the second case, Peter, behavioral issues are the most prevalent. The third case of Jeanette describes symptoms that can be seen as a depressive reaction, where a change in mood and behavior has occurred following negative experiences. Another explanation for discrepant findings of interrater reliability for V4 could be that the scoring instructions are unclear or too broad. For V-RISK-10, interrater reliability for V4 was good, with ICC value of 0.70 for single measures and 0.83 for average measures [29]. It is possible that this item is harder to score for youth than it is for adults. The pilot study [32] and the V-RISK-10 study [29], which both found higher ICC values for V4, were conducted in acute psychiatric inpatient units only. In this study, interrater reliability was not higher for the acute psychiatric units as compared to the other youth facilities, so the differences cannot readily be explained by differences in types of institutions. The V-RISK-10 study was conducted in a naturalistic setting, and the discrepancies between these findings could imply difficulties in scoring this item for a vignette as compared to in-person cases. However, another V-RISK-10 study assessing interrater reliability through a case vignette design with 15 vignettes and eight raters yielded similar results [13].

Interrater reliability for item V7 (Suspicion) was poor across all groups. The description of V7 is largely based on exhibited behavior, which can be difficult to judge from a vignette without relying on behavioral observations. In the V-RISK-Y pilot, however, interrater reliability for this item was good, with an ICC estimate of 0.76 [32].

Interrater reliability is moderate for V10 (Stress exposure) for all types of facilities, except for the CPS institutions where it is poor. Further, interrater reliability for V10 is lower for professions other than psychologists and physicians. It is possible that these differences could reflect that staff groups have different ways of assessing stressful situations. Physicians and psychologists in the psychiatric units are typically responsible for treatment, whereas other staff groups are more present in the institutional environment outside of treatment sessions. These findings could also be impacted by most staff from the other professions group being from the CPS institutions. Possibly, staff in psychiatric units for youth have different ways of assessing stressful future situations as compared to CPS staff. Differences in ways of thinking about future stress may for instance be due to institutional characteristics, where the relatively closed environment of an inpatient unit might be perceived as mitigating stressful situations as compared to a more open residential setting. Interrater reliability for this item was good (ICC = 0.76) in the pilot study [32], which was conducted in a psychiatric unit.

For V12 (User perception), one of the items novel to V-RISK-Y, interrater reliability was poor to moderate for all groups. This finding is comparable to the pilot study, where ICC for this item was 0.35 [32]. This item was included in V-RISK-Y based on findings that patients’ own perception of violence risk is significantly associated with actual risk [47]. Patients’ own perception is not commonly included in existing screenings and assessments of violence risk [e.g. 48], and potentially represents a new way of thinking about risk assessments which may make it difficult to score. Further, this item is challenging to score based on the provided case vignettes, where there was little information about the youth or parents’ perceived risk of violence. A study conducted in a naturalistic setting, where the youth and their guardians could be asked about risk perception, might yield better interrater reliability for this item.

In the V-RISK-10 study where interrater reliability was assessed in a naturalistic setting, some items were found to have poor interrater reliability, including item V7 (Suspiciousness) and V10 (Future stressful situations). However, in subsequent research on predictive validity of V-RISK-10, items with low interrater reliability were still found to have high predictive validity [e.g. 49, 50], and the items are kept in the instrument. Before deciding what to do with items with low interrater reliability, results from this study must be seen in relation to findings from ongoing efforts to validate V-RISK-Y, and be compared to items’ contribution to predictive validity of the instrument.

Limitations and future research.

There are some limitations in the study design that may have impacted results, which should be considered in future research on psychometric properties of V-RISK-Y. The case vignette design allows to control for the information provided to score each case. However, while efforts were made to design vignettes resembling clinical cases, a case vignette design does not reflect a naturalistic setting. Only a narrow range of scenarios are represented in the included vignettes, which do not cover complexities and diversity of real-life contexts. These limitations impact the generalizability of the findings to settings outside of theoretical case scorings. Case vignettes are commonly used in violence risk assessment trainings for skill development [e.g. 51, 52]. It is possible that the cases would be better suited for a training purpose than realistic assessment of interrater reliability. Nevertheless, the good interrater reliability found for the sum score and the Low-Moderate-High risk level lend support for continuing research efforts on the current version of V-RISK-Y. It would also be of interest to conduct a naturalistic study of interrater reliability as was done for V-RISK-10.

Demographic information about participating staff was not collected in the present study. Thus, findings cannot be interpreted in relation to demographic variables such as work experience, age or sex. These variables should be included in future research to enable more distinguished comparisons between included staff groups, which particularly would be of interest because V-RISK-Y is developed to be easy to use for all clinical staff.

The design of this study included only three cases and a high number of participants. In studies with low between-rater variance, which may be the case when all participants are in similar work environments, precision of ICC is facilitated by a high number of raters and a low number of cases [53]. It is likely that the large confidence intervals for the estimated ICC values and the inability to compute ICC for some items in the stratified analyses on profession reflects the design of a high rater to case ratio. A study with a different setup, where more cases are scored by fewer participants, could mitigate this issue and allow for further assessment of discrepancies in interrater reliability found in stratified analyses.

In this study, cases were scored individually by the participants. The recommendation for V-RISK-Y is interdisciplinary rating when possible. Further research should assess whether interdisciplinary versus individual scoring influences the psychometric properties of V-RISK-Y. In this study, as well as in the pilot, “Don’t know” scores are coded as 1 and included in the ordinal scale of the single items, based on findings on V-RISK-10 showing that don’t know scores should be counted toward risk [54]. To date, there is no research exploring whether the same argument holds true for V-RISK-Y, which should be assessed in future studies.

## Conclusions

Results from this initial study on interrater reliability for V-RISK-Y are promising. While poor interrater reliability was found for some of the risk items, the overall agreement on sum of present risk factors and risk level is high. Findings indicate acceptable interrater reliability for V-RISK-Y across different types of youth facilities where the objective of identifying violence risk commonly occur, namely acute psychiatry, outpatient psychiatry, and child protective services. Given limitations in the study design, findings should be cautiously interpreted, and generalizability to naturalistic settings cannot be readily assumed. This is the first interrater reliability study of the current version of the V-RISK-Y, and an important step in establishing the psychometric properties of this instrument. Research on V-RISK-Y is still in its early stages, and there is a need for further studies to assess its psychometric properties in naturalistic settings.

## Data Availability

The datasets analyzed in the current study are not publicly available and cannot openly be shared due to privacy laws and restrictions in ethical approval. Data are available from the authors upon reasonable request.

## Appendix: case vignettes

### Case 1: Farhad [15]

Farhad is referred for assessment of behavioral disorder. He struggles with schoolwork and in social interactions with peers. As a young child (6–8 years), he kicked and hit his peers, without anyone getting physically hurt. At 13, he attacked a teacher who suggested he received special education, resulting in the teacher sustaining a concussion. He is easily upset, particularly if he feels misunderstood, devalued, or struggles to express himself. At home, he spends most of his time in front of the computer. During initial contact, he seems disinterested in engaging in conversation, and is annoyed when staff asks him questions. Peers are uneasy around him. He does not understand that others get upset when he hits or kicks them, and says it’s their fault for treating him unfairly.

### Case 2: Peter [17]

Peter struggles to adhere to rules and structure and demonstrates lack of respect for authorities. In school, he once lifted a teacher out of the classroom and locked the door. At home he is aggressive and destructive, and his parents often give in to what he wants out of fear that he will destroy things or hurt them. Peter describes his parents as weak. He does not get along with his peers, but has a few younger friends. He stays out late, and drinks alcohol on the weekends. He recently physically assaulted someone for calling him gay. He wants to move out from his parents house and become rich.

### Case 3: Jeanette [15]

Jeanette has always been ambitious in school, but lately she’s been reluctant to go to school and her grades have dropped. Upon her parents’ separation one year ago, Jeanette started spending less time at home and started going to the mall. A few weeks ago she got drunk with her friends, and was the victim of an attempted rape. She did not tell her parents about this incident. She is normally good with her younger siblings, but recently she yelled at her little brother when he entered her room and threatened to hit him. Her mother has noticed that Jeanette has started self-harming by cutting her wrists. In the initial contact, she seems resigned, and lets her mother answer for her.
